# Reasons for delay in decision making and reaching health facility among obstetric fistula and pelvic organ prolapse patients in Gondar University hospital, Northwest Ethiopia

**DOI:** 10.1186/s12905-017-0416-9

**Published:** 2017-08-22

**Authors:** Mulat Adefris, Solomon Mekonnen Abebe, Kiros Terefe, Abebaw Addis Gelagay, Azmeraw Adigo, Selamawit Amare, Dorothy Lazaro, Aster Berhe, Chernet Baye

**Affiliations:** 1 Department of Obstetrics and Gynecology, University of Gondar, P.O.Box 196, Gondar, Ethiopia; 2 Institute of Public Health, University of Gondar, Gondar, Ethiopia; 3 UNFPA Country office, Addis Ababa, Ethiopia

## Abstract

**Background:**

Obstetric fistula and pelvic organ prolapse remain highly prevalent in sub-Saharan Africa, where women have poor access to modern health care. Women having these problems tend to stay at home for years before getting treatment. However, information regarding the reasons contributing to late presentation to treatment is scarce, especially at the study area.

The objective of this study was to assess the reasons whywomen with obstetric fistula and pelvic organ prolapse at Gondar University Hospital delay treatment.

**Method:**

A hospital based cross-sectional study was conducted among 384 women. Delay was evaluated by calculating symptom onset and time of arrival to get treatment at Gondar University Hospital. Regression analysis was conducted to elicit predictors of delay for treatment.

**Result:**

Of the total 384 participants, 311 (80.9%) had pelvic organ prolapse and 73(19.1%) obstetric fistula. The proportion of women who delayed treatment of pelvic organ prolapse was 82.9% and that of obstetric fistula 60.9%. Fear of disclosing illness due to social stigma (AOR = 2; 1.03, 3.9) and lack of money (AOR = 1.97; 1.01, 3.86) were associated with the delay of treatment for pelvic organ prolapse,while increasing age (AOR =1.12; 1.01, 1.24) and divorce (AOR = 16.9; 1.75, 165.5) were were responsible for delaying treatment forobstetric fistula.

**Conclusion:**

A large numberof women with pelvic organ prolapse and obstetric fistula delayed treatment. Fear of disclosure due to social stigma and lack of moneywere the major factors that contributed to thedelay to seek treatment for pelvic organ prolapse,while increasing age and divorce were the predictors for delaying treatment for obstetric fistula.

## Background

About 20–30 women develop serious morbidities for every maternal death [1, 2]. These morbidities include pelvic organ prolapse (POP) and obstetric fistula (OF). POP is defined as a down ward descent of pelvic structures and parity as well as advanced age were reported to be the most important risk factors [3, 4].

OF, the most devastating morbidity [2, 5] caused by prolonged and obstructed labor, is defined as an abnormal hole between a woman’s vagina and bladder or rectum through which urine or stool continually leak [1]. POP and OF are major public health problems in developing countries [6]. Worldwide POP occurs in about 316 million women (9.3% of all females) [7] .However, the prevalence of POP in low and middle-income countries is reported to be 19.7% (range 3.4%–56.4%) [8]. Apilot study conducted at Dabat district, Ethiopia, revealed the prevalence of symptomatic pelvic organ prolapse as 6.3%, whereas anatomic prolapse stage (II-IV) was 162(55.1%) [9].

Similarly,each year, between 50,000 to 100, 000 women worldwide develop OF, and an estimated 2–3 million have OF globally [10, 11]. A meta-analysis in 19 sub-Saharan African countries noted that Ethiopia and Uganda have the largest number of women withsymptoms of OF. In Ethiopia,about 110,000 women in have OF. The Ethiopian Demographic and Health Survey data noted a life time prevalence rate of10.6 per 1000 OF cases among women who had ever given birth [12, 13].

POP and OF greatly affect women’s quality of life and result in physical, social, psychological, sexual and economic problems [14] Understanding the effects of POP and OF in women’s experiences or quality of life is important to increase understanding of the condition and encourage early treatment seeking behavior A qualitative study done among POP cases in Sweden showed that delay in seeking health care was influenced by absence of information, blaming oneself, feeling ignored by the doctor and adapting to successive impairment [15].

Most women in Ethiopia do not seektreatment for POP and OF despite the availability of treatment facilities for both conditionsin the country [2]. Studies identified that women with POP and OF tend to stay at home for years before getting treatment [16, 17]. However, barriers toearly presentation to treatment and the reasons for the delay in seeking treatment is not well known. Hence, this study aimed to identify factors contributing to thedelay in decision making to seek treatment among POP and OF patients at Gondar University Hospital, northwest Ethiopia.

## Methods

### Study setting

Gondar is a densely populated historical city of the Amhara Region (capital of Ethiopia in seventeenth century) with an estimated urban population of 254,420,(120,569 male and 133,851 female). The study was conducted at Gondar University Hospital which is located in Gondar city, the northwestern part of the country. The hospital was established in 1956 and currently has more than 500 beds; of these, 70 beds and two major operation theatre are dedicated toOF and POP patients; since 2011 the treatment cost is covered by the hospital and the United Nation Population Fund (UNFPA).

### Study design and population

A hospital-based cross-sectional study was conducted to assess delays in seeking treatment. The participants population were all women admitted for POP and OFto the Fistula Center of the hospital from Jan 2015 to Jan 2016

### Study variables

- Dependent variable: Delay in deciding to seek treatment for POP and OF. Socio-demographic characteristics of women (age, religion, ethnicity, marital status, educational status), household income, number of children, failure to recognize signs of complication, failure to perceive severity of illness, decision making power, distance from health facility, health care cost, and means of transportation were collected as independent variables. In addition, the questionnaire included, duration of POP and OF, lifestyle (blaming oneself, feeling ignored by the community, living with family, ureteral reconstruction, distance from health facility, fleeing bad smell, early marriage, experience about health system, health care cost), clinic attendance, cultural factor (Value of women health, decision making power, awareness about the problem). Fistula cases who sought health care within 3 months were classified as “good”, while above 3 months were considered as “poor” or delayed. POP caseswho stayed until12 months and above to get treatment wereconsidered as “delayed”.

### Data collection

Data were collected by interviewing all eligible participants, using a pretested structured questionnaire. We assessed using the two delay model approach which recognizes the different factors women face in achieving timely and effective medical care needed for OF and POP [18]. Data was collected for one year by four trained Bachler of science nurses.

### Quality control methods

The study tools were initially developed in English and then translated into Amharic and back into English to ensure consistency and accuracy in the phrasing of questions. The interview was conducted in Amharic after questioner was pretested on ten women at different hospital located in community culturally closely affiliated to Gondar. Training was given to data collectors and a strict supervision was made by the research team, and 5% of the collected data was checked daily for completeness and consistency.

### Data processing and analysis

The data was coded, double entered, and cleaned using Epi-info version 7and exported to STATA version 12 for analysis. Delay in decision to seek treatment was evaluated by calculating symptom onset and time of decision to seek treatment. Both univariate and bivariate analysis were done to describe frequency, percentage and themean; multivariable regression was carried out e to see the presence and strengths of association.

Independent variables were selected based on the literature, and a cut of point at *p*-value ≤0.20 in the bivariate test was included in the multivariable logistic regression model. Multi-collinearity effect was also checked for the full model. Informed consent was obtained from the study participants, and the study was approved by the IRB of the University of Gondar.

## Result

### Socio-demographic and economic characteristics

A total of 384 eligible women took part in the study. Of these, 311 (80.9%) were POP and 73 (19.01%) OF cases.. The mean age of the participants was 42.57 years (SD ± 13.1 years) with a range of 12 to 85 years old. The mean age of POP cases was 45.27 (SD ± 12) years and that of OF 31.1 (SD ± 10.3) years (Table 1). Most (78%) of the participants had given birth at home. The proportion of still births among OF cases was high 54 (73.97%). The socio-demographic and lifestyle characteristics of the participants were stratified by POP and OF (Table 1).

**Table 1:** The socio-demographic and life style characteristics of the study participants stratified by POP and Fistula case at the Hospital of Gondar University

Variable	Total n(%)	POP n(%)	Fistula n(%)
Age in Years			
12–24	28 (7.3)	9 (2.9)	19 (26)
25–34	65 (16.9)	35 (11.3)	30 (41.1)
35–44	126 (32.8)	113 (36.3)	13 (17.8)
45 and above	165 (42.9)	154 (49.5)	11 (15.1)
Resident			
Urban	63 (16.5)	45 (14.5)	19 (26.1)
Rural	320 (83.5	266 (85.5)	54 73.9)
Age at 1st Delivery			
Less than 18 years old	238 (61.9)	204 (65.6)	34 (46.6)
18 years and older	146 (38.02)	107 (34.4)	39 (53.4)
Marital Status			
Single	40 (10.4)	29 (9.32)	11 (15.1)
Married	200 (52.1)	165 (53.1)	35 (47.9)
Divorced	86 (22.4)	63 (20.3)	23 (31.5)
Widowed	53 (13.8)	51 (16.4)	2 (2.74)
Separated	5 (1.30)	3 (0.96)	2 (2.74)
Education Status			
Unable to read and write	333 (86.7)	283 (91)	50 (68.5)
Grade 1 to 7	37 (9.6)	23 (7.4)	14 (19.2)
Grade 8 to 12	12 (3.13)	5 (1.61)	7 (9.59
Deploma and above	2 (0.52)	0 (0.00)	2 (2.74)
Parity			
No child	7 (1.8)	4 (1.3)	3 (4.1)
1 to 2	71 (18.5)	35 (11.2)	36 (49.3)
3 to 6	163 (42.4)	137 (44.1)	26 (35.6)
7 to 10	128 (33.3)	121 (38 9)	7 (9.6)
11 to 15	15 (3.9)	14 (4.5)	1 (1.4)
Still birth			
Yes	116 (30.2)	62 (19.9)	54 (73.9)
No	268 (69.8)	249 (80.1)	19 (26.1)

The mean ages at first marriage among OF and POP cases were 14.42 (±SD 2.9) years and 13.3 (±3.4 SD) years,respectively. About 89.6% of the OF and 97.6% of the POP cases were married before celebrating their 18th birth days. The proportion of women who gave the first birth before the age of 18 years were 46.6%. Of these, 27.4% OF patients gave birth before the age of 15,while 65.6% POP cases gave the first births before the age of 18. The mean number of parity of POP was 6 (SD ± 2.7) and 3.2(SD ± 2.7) for OF. Overall, 70.4% of POP and 26% of OF cases were grandmultipara (had 5 or more births). About 44.9% OF and 16.1% POP reported that they were isolating themselves (Fig. 1).

About 88.7% of the POP patients had stage 3 pelvic organ prolapse (Fig. 2). More than 95% of OF and POP cases were economically dependent. About half (49%) of OF disclosed their problem to their family, and 99.7% of OF cases mentioned the smell of urine and faeces as the reasons of the community for not willing to live with them.

### Proportion of delay in seeking treatment

The proportion of women who delayed for POP was 82.6% [95% CI: 78.4%, 86.8%], and that of OF was 63% [95% CI: 51.7%, 74.3%]. The mean length of delay for POP and OF were 85.8 and 48.3 months with a range of 1–348 months, respectively.

Descriptively, the participants said that unavailability of roads (91.6%), lack of transportation (87.7%), distance from health facility (requiring 2 days or more to reach health facility) (35.5%), and walking (82.2%) were the main barriers to seeking early treatment for POP and OF cases.

### Determinants of delays in seeking treatment for pelvic organ prolapse and obstetric fistula

In the multivariate analysis, factors that were significantly associated with delays of seeking treatment among POP were fear of disclosure and lack of money. Women who had fear of disclosure were two times (AOR = 2; 1.03,3.9) more likely to delay treatment for POP as compared to those who had no such fear. Women who mentioned lack of money as the reason for delay were two times (AOR = 1.97; 1.01,3.86) more likely to delay seeking treatment for POP as compared to those who didn’t mention lack of moneyas the reason for the delay (Table 2).

**Table 2:** Multivariable logistic regression analysis showing the relationship between decision delay to seek professional health care and factors associated among pelvic organ prolapse patients in Gondar referral Hospital of Gondar, Northwest Ethiopia

Variable	Yes n(%)	Crud Odds Ratio [95% CI:]	Adjusted Odds Ratio [95% CI:]
Age in Years			
12–24	5 (55.6)	1.00	1.00
25 to 34	31 (88.6)	6.2 [1.16–33.17]	0.33 [0.07–1.62]
35 to 44	95 (84.1)	4.2 [1.03–17.26]	1.48 [0.39–5.71]
45 to 54	68 (81.9)	3.6 [0.87–15.13]	1.14 [0.47–2.75]
55 and above	58 (81.7)	3.6 [0.84–15.15]	1.16 [0.47–2.83]
Education status			
Unable to read and write	236 (83.4)	1.00	1.00
Grade 1 to 7	17 (73.9)	0.57 [0.21–1.50]	1.71 [0.16–17.68]
Grade 8 to 12	4 (80.0)	0.79 [0.087–7.28]	1.12 [0.0913.75]
Marital Status			
Single	22 (75.9)	1.00	1
Married	139 (84.2)	1.70 [0.66–4.39]	1.66 [0.59–4.64]
Divorced	53 (84.1)	1.68 [0.57–4.99]	1.56 [0.48–5.12]
Widowed	41 (80.4)	1.30 [0.43–3.90]	1.07 [0.30–3.79]
Separated	2 (66.7)	0.63 [0.05–8.12]	0.51 [0.03–8.07]
Lack of support			
No	202 (81.4)	1.00	1.00
Yes	55 (87.3)	1.56 [0.69–3.51]	1.41 [0.60–3.33]
Fear of disclosure			
No	167 (80.7)	1.00	
Yes	90 (86.5)	1.55 [0.85–2.83]	2.00 [1.03–3.90]*
Use modern Medicine			
Yes	121 (79.1)	1.00	
No	136 (86.1)	1.64 [0.90–2.96]	1.91 [0.99–3.67]
Do you isolate your self			
No	212 (81.5)		
Yes	45 (88.2)	0.59 [0.24–1.46]	0.71 [0.27–1.86]
Low Income			
No	119 (77.8)	1.00	
Yes	138 (87.3)	1.97 [1.08–3.61]	1.97 [1.01–3.86]*

*= *p*-value ≤0.05

After adjusting for a number of important covariates, the multivariable logistic regression analysis showedthat OF patients who were divorced had a significant association with delay in seeking treatment compared to singles. The odds of delay in seeking treatment were 16 times higher among divorced than never married women (AOR = 16.9; 1.75,165.5). The multivariable logistic regression analysis showed that when a person got older by one year, the odds of delay in seeking treatment for OF increased by 12% (AOR =1.12; 1.01, 1.24) (Table 3).

**Table 3:** Multivariable logistic regression analysis showing the relationship between decision delay to seek professional health care and factors associated among obstetrics fistula patients in Gondar referral Hospital of Gondar, Northwest Ethiopia

Variable	Delayed n(%)	Crude OR [95% Conf. Interval	Adjusted OR [95% Conf. Interval
Age		1.09 [1.02–1.16]	1.12 [1.01–1.24]*
Marital Status			
Single	4 (36.4)	1.00	1.00
Married	17 (48.6)	1.65 [0.41–6.67]	1.28[0.24–6.72]
Divorced	25 (92.6)	21 [3.29–145.2]	16.9 [1.75–165.5]*
Distance was reason for delayed			
No	31 (60.8)	1.00	1.00
Yes	15 (68.1)	1.38 [0.47–3.98]	0.26 [0.05–1.41]
Economic status			
Good	23 (50)	1.00	1.00
Poor	23 (85.2)	5.75 [1.71–19.3]	3.49 [0.56–21.7]
Parents alive			
Yes	31 (56.4)	1.00	1.00
No	15 (83.3)	3.87 [1.01–14.9]	0.33 [0.03–3.44]
Availability of treatment center			
No	39 (60.9)	1.00	1.00
Yes	7 (77.8)	2.24 [0.43–11.7]	3.68 [0.31–44.4]
Fear of small			
No	36 (58.1)	1.00	1.00
Yes	10 (90.9)	7.22 [0.89–59.9]	5.59 [0.41–76.1]
Disclosure of the problem			
No	19 (61.3)	1.00	1.00
Yes	27 (64.3)	0.89 [0.34–2.29]	0.26 [0.05–1.40]
Afraid of losing social value			
No	38 (59.4)	1.00	1.00
Yes	8 (88.9)	5.47 [0.65–46.4]	14.91 [0.98–225.7]

*= *p*-value ≤0.05

## Discussion

The aim of this study was to assess the proportion of women with POP or OF who delayed seeking treatment and to identify factors associated with the delays (delay to decide to seek care and treatment (first delay) and delay to reach treatment center (second delay). In this institution- based study, we found a high proportionof delay in seeking treatment for both POP and OF participants. Factors associated with the in seeking treatment for POP were fear of disclosure and lack of money, and for OF marital status and age.

In this study, it was noted that the proportion of women who delayed seeking treatment for POP (82.6%), and OF (63%) was high. Similarly, the mean length of delay for POP was 85.8 months (SD± 80.2), which is higher than the report from Israel (41.2 months with SD ± 39.5) [19]. However, the mean length of delay for OF (48.3 months) was in line with a similar study done in Israel (47.6 months) [19]. Due to social stigma attached to these problems, women with POP and OF usually feel embarrassed, isolate themselves,and this in turn results in different social and psychological consequences. These also make women not to disclose their problems to their relatives and keep them secret and thereby delay seeking treatment [20, 21]. In this study, it was noted that women who had fear of disclosure were two times more likely to delay seeking treatment for POP compared to those who had no fear of disclosure.

Financial constraint was one of the reasons for delaying seeking treatment for POP. In this finding, women who mentioned lack of money as a reason for the delay were nearly two times more likely to delay seeking treatment for POP as compared to those who didn’t mentionlack of money as the reason for the delay. Women with low or no income were not able to cover the health service expenses (medical and surgical), transport, foodand other indirect costs. Most women, especially rural women in Ethiopia are not economically empowered [22]A systematic review also indicated that financial problem was the most frequently mentioned reason for delaying to seeking treatment [23]. As POP is not a lifethreatening condition, many patients don’t want to seek treatment even if they are symptomatic. However, early treatment improves patient quality of life by improving the social, psychological, physical and sexual life of the women.

In this study, it is identified that fistula patients who were divorced were more likely to delay seeking treatment for OF than unmarried ones. Most fistula patients experienced stigma from their husbands, families and the community. A systematic review identified that socio- cultural factors were the second most frequent barriers to early treatment seeking. Different studies noted that more than 50% of women with OF were divorced [16, 23]. This implies that social problems, including social stigma and divorce can prevent women from getting early treatment [24].

In our work, age was found to have a significant association with delays to seek treatment for OF. The older the women the longer the delay to seek treatment for OF. Older women may believe that the symptom or the problem is a part of aging and tend to become reluctant to seek medical help.

The limitation of our study is that, some of the information was based on self-report which make it subject to recall bias. Hence, a longitudinal research is needed to assess the relationship amongvariables over time. Even though all patients were getting treatment during data collection, the sample size, especially the number of fistula cases was too small for us to do a rigorous and detailed analysis on the various risk factors.This study can be regarded as an eye opener for studies on POP and OF in Ethiopia and has clearly indicated the need for further detailed research with sufficiently large sample size. Another limitation of this study is its across-sectional design which might not show temporal relationships and thus making the observed associations not necessarily causal.

## Conclusion

A large number of women with POP and OF delayed seeking treatment. Fear of disclosure due to social stigma and financial constraints were the major factors that contributed to the delays of seeking treatment for POP,while old age and divorced marital status were the major predictors of delays in seeking treatment for OF.These social problems contribute todelays in seeking treatment in two ways; one is their negative impact on women’s economic status, and the other is that is due to the problem no one offers to continually support and encourage the woman to seek treatment.

### Recommendations

The authors recommended that the government, health professionals, communities, families, and husbands should play a role in reducing delays in seeking treatment for POP and OF. The government needs to increase the geographic and economic accessibility of the services. Health professionals, more importantly community health extension workers, should address cognitive accessibility to the community and families about the problems, and to identify cases and facilitate referral to nearby treatment centers. Creating awareness in the community and families about the real nature of the problem will help POP and OFpatients to get the necessary support from the community and their families, and this will in turn reduce delay in treatment and social stigma.

## Abbreviations

OF: Obstetric Fistula
POP: Pelvic organ prolapse
